# Application of retrograde distal perfusion via posterior tibial artery in venoarterial extracorporeal membrane oxygenation: A retrospective single-center study

**DOI:** 10.1016/j.xjtc.2026.102271

**Published:** 2026-02-04

**Authors:** Kun Li, Dandan Ding, Zongwei Gao

**Affiliations:** a Department of Critical Care Medicine, Linyi People's Hospital, Linyi, China; b Gynecology Department, Linyi Cancer Hospital, Linyi, China

**Keywords:** retrograde distal perfusion, antegrade distal perfusion, posterior tibial artery, lower extremity ischemia, distal perfusion catheter, VA-ECMO

## Abstract

**Objective:**

This study aimed to evaluate the feasibility of retrograde distal perfusion via the posterior tibial artery (PTA) in patients receiving venoarterial extracorporeal membrane oxygenation (VA-ECMO).

**Methods:**

A retrospective single-center study was performed on the clinical data of 48 patients who underwent VA-ECMO and were administered retrograde distal perfusion between April 2023 and July 2025. The surgical technique for cannulation via the PTA was described, and this perfusion approach was compared with antegrade distal perfusion.

**Results:**

All 48 cases of retrograde distal perfusion were successfully achieved via surgical cannulation. The cannula size was mainly 6 Fr, and the cannulation time was 18.98 ± 4.10 minutes. Imaging studies confirmed that retrograde blood flow could ascend to the superficial femoral artery and deep femoral artery. Compared with the antegrade approach (comprising 93 percutaneous and 23 surgical cases), the retrograde method achieved a significantly greater cannulation success rate than the percutaneous approach (95.83% [46/48] vs 53.76% [50/93], *P* < .05) and a significantly lower incidence of infection than the surgical approach (0.00% [0/48] vs 34.78% [8/23], *P* < .05). No significant difference was observed in survival rates among the groups.

**Conclusions:**

In VA-ECMO, retrograde distal perfusion via the PTA is a feasible strategy. Like antegrade distal perfusion, it effectively mitigates lower extremity ischemia.

**Figure:**
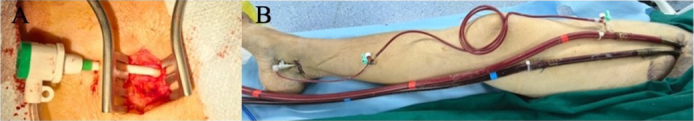
Retrograde distal perfusion posterior tibial artery.

**Central Message**

This study aimed to evaluate the feasibility of retrograde distal perfusion via the posterior tibial artery (PTA) in patients receiving venoarterial extracorporeal membrane oxygenation (VA-ECMO).

**Perspective**

This study provides a novel perspective that retrograde DPC cannulation via PTA could be a preferred alternative for patients with slender femoral arteries, where antegrade perfusion is not feasible.

In venoarterial extracorporeal membrane oxygenation (VA-ECMO) with peripheral cannulation, the femoral artery and femoral vein are commonly selected as access sites. However, femoral artery cannulation frequently carries the risk of inducing acute limb ischemia (ALI) in the ipsilateral extremity, which has become a serious complication of VA-ECMO.^1^ Typically, the placement of a distal perfusion catheter (DPC) in the ipsilateral limb is the primary method for preventing and alleviating ALI. Currently, most reports^2^^,^^3^ have adopted the method of antegrade distal perfusion via cannulation of the superficial femoral artery (SFA). In contrast, the use of a DPC placed in the posterior tibial artery (PTA) to achieve retrograde distal perfusion has been rarely documented—despite this being a feasible and relatively novel approach. This perfusion method remains underrecognized, and relevant research on its application is extremely limited.

This study will further introduce the method of retrograde distal perfusion via PTA in VA-ECMO, including detailed surgical procedures and methods for verifying the effectiveness of this approach using ultrasound and other imaging modalities. Furthermore, we will conduct the first comparative analysis, to our knowledge, between retrograde perfusion and antegrade perfusion, to further explore the feasibility of the retrograde approach and provide more convincing data.

## Methods

### Subjects and Methods

#### Study subjects

All VA-ECMO cases performed at our extracorporeal membrane oxygenation (ECMO) center from April 2023 to July 2025 were selected, with cannulation strategies including both percutaneous and surgical approaches. The study protocol was approved by the Science and Technology Ethics Committee, Linyi People's Hospital (approval number: 202509-H-005), and the requirement for obtaining informed consent was waived. Inclusion criteria included adult patients who received VA-ECMO with either retrograde or antegrade DPC placement. Exclusion criteria were patients with a VA-ECMO support duration of less than 72 hours.

#### Study methods

The surgical technique for PTA cannulation and the decannulation procedure for DPC have been detailed previously.^4^ A comparative analysis was performed between retrograde and antegrade distal perfusion (including both percutaneous and surgical cannulation subgroups).

#### Statistical methods

Statistical analyses were performed using SPSS 27.0 software (IBM Corp). Continuous variables are presented as mean ± standard deviation ($\bar{x}$ ± s). On the basis of normality distribution assessment, one-way analysis of variance or Kruskal-Wallis test was used for intergroup comparisons. Categorical variables are expressed as frequencies and percentages, ie, n (%), with intergroup comparisons performed using the χ^2^ test or Fisher exact test as appropriate.

## Results

### Surgical Techniques and Verification

#### Surgical technique

**Figure 1:**
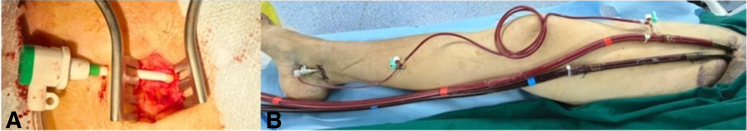
DPC placement via the PTA. A, Cannula placement in the PTA. B, Retrograde distal perfusion via the PTA cannula connected to the arterial branch of the ECMO circuit. *DPC*, Distal perfusion catheter; *PTA*, posterior tibial artery; *ECMO*, extracorporeal membrane oxygenation.

The ipsilateral medial ankle was exposed by external rotation of the leg, and a transverse incision of 5 cm in length was just made posterior to the medial malleolus. Dissection was performed layer by layer until the PTA was identified, followed by circumferential isolation of the artery. The artery could be suspended with a silk suture to facilitate puncture. Finally, an arterial catheter was inserted using the Seldinger technique and connected to the arterial limb of the ECMO circuit (Figure 1). The incision was then sutured closed, with blood flow monitored via a flow monitoring system.

**Figure 2:**
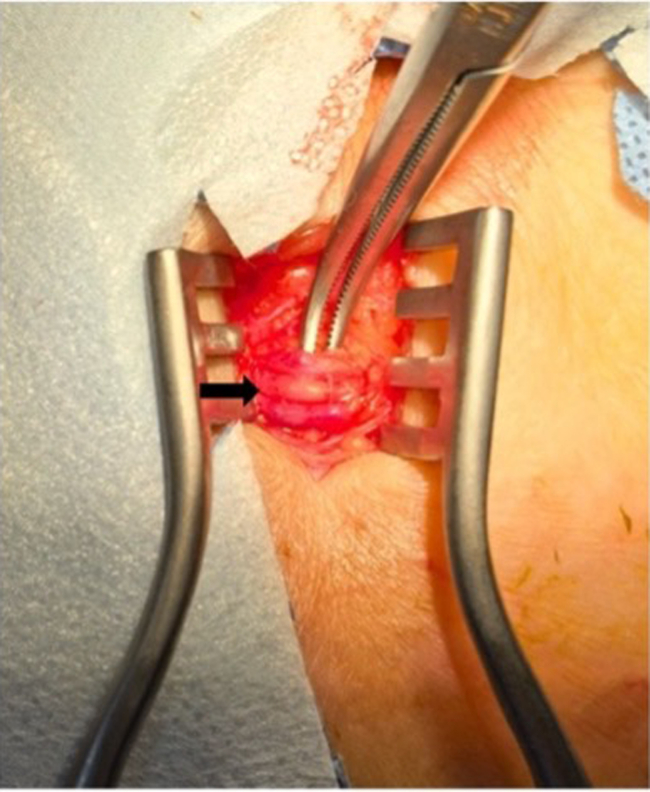
The PTA and its 2 venae comitantes. The PTA is indicated by the *arrow*, with veins located on both sides. *PTA*, Posterior tibial artery.

**Figure 3:**
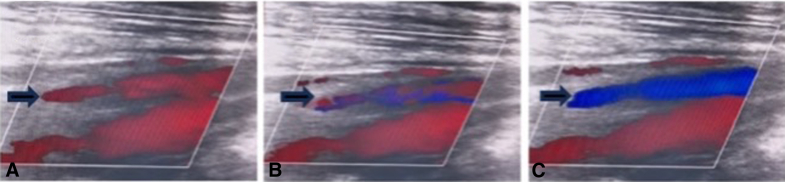
Color Doppler ultrasonographic imaging of the SFA. The SFA is indicated by the *arrow*, with the femoral vein situated inferiorly. Ultrasonography revealed bidirectional flow within the SFA: (A) The flow direction in the SFA was consistent with that in the femoral vein, indicating retrograde arterial flow; (B) transitional moment of bidirectional flow within the SFA; (C) distinct flow directions in the SFA and the femoral vein. *SFA*, Superficial femoral artery.

Surgical key points include the following: (1) The PTA is superficially located, approximately 2 cm posterior to the medial malleolus. (2) The PTA is accompanied by 2 venae comitantes, with the artery situated between the 2 veins (Figure 2). (3) After catheter insertion, backflow of blood should ideally be observed to confirm true lumen access. Postoperative bedside color Doppler ultrasonography may be performed for verification (Figure 3).

**Figure 4:**
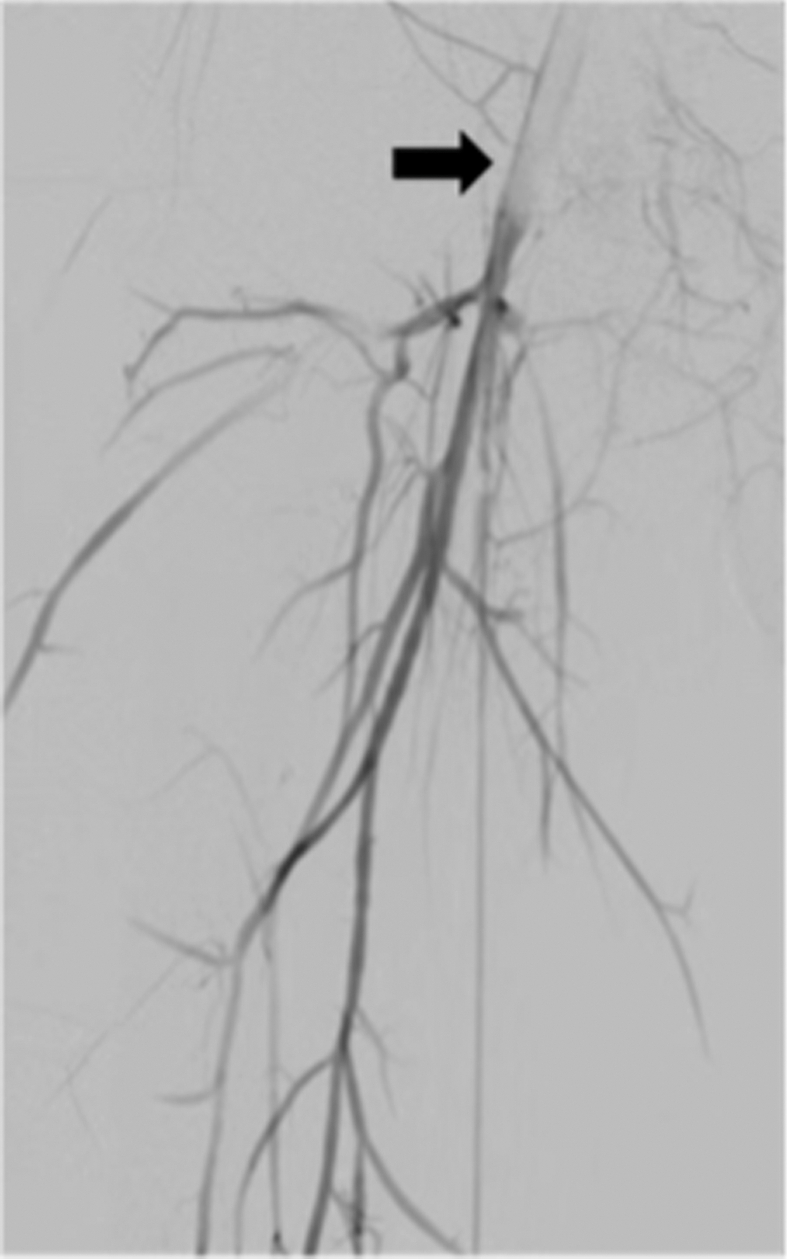
Retrograde distal perfusion angiography. Contrast medium was injected via the PTA cannula and flowed retrogradely upward, with clear opacification of distal vessels including the SFA and DFA. The *arrow* indicates the ECMO femoral artery cannula, showing a filling defect. *PTA*, Posterior tibial artery; *SFA*, superficial femoral artery; *DFA*, deep femoral artery; *ECMO*, extracorporeal membrane oxygenation.

In 1 ECMO case, contrast angiography was performed via the DPC of the PTA. Imaging revealed clear opacification of the SFA, deep femoral artery (DFA), and distal blood vessels. This finding confirmed that retrograde distal perfusion is fully feasible (Figure 4).

#### Decannulation

The PTA cannula was removed, and hemostasis was obtained by direct pressure for at least 30 minutes.

### Data Collection and Analysis

#### General data

From April 2023, to July 2025, a total of 218 VA-ECMO cases were completed, comprising 173 percutaneous cannulations and 45 surgical cannulations. All patients in our center underwent prophylactic DPC placement. After percutaneous cannulation, either antegrade or retrograde DPC was placed; after surgical cannulation, antegrade DPC was placed under direct vision. Patients with an ECMO support duration of less than 72 hours and pediatric patients were excluded. Finally, 48 cases with retrograde distal perfusion, 93 cases with percutaneous antegrade distal perfusion, and 23 cases with surgical antegrade distal perfusion were included in the analysis.

#### Retrograde distal perfusion data

Retrograde distal perfusion via the PTA was performed in 48 patients. All cannulations were surgically placed for prophylaxis, with 6-Fr catheters primarily used. Two patients developed lower-limb ischemia, which was successfully managed by contralateral ECMO cannulation and common femoral artery repair. For the diagnosis of ALI, patients who were conscious were assessed mainly on the basis of subjective symptoms: the possibility of ALI should be suspected when numbness, pain, or other relevant sensations develop and progressively aggravate. For patients who are unconscious, the diagnosis was primarily made by comparison with the contralateral lower extremity; ALI should be considered if the limb on the side of ECMO arterial cannulation exhibits pallor, cool skin temperature, mottling, and other abnormal changes.

Cannulation success rate was 95.83%, with failures attributable to severe atherosclerosis (n = 1) and inadvertent cannulation into a PTA dissection (n = 1). Low rates of hematoma and infection were observed. Survival rate was 72.92% (see Table 1).

**Table 1:** Clinical data of 48 patients with retrograde distal perfusion via the PTA

Item	Data
Perfusion approach	
Retrograde via PTA	48 (100.00)
Cannulation timing	
Prophylaxis (<6 h)	48 (100.00)
Cannula size, Fr	
6	40 (83.33)
8	6 (12.50)
5	2 (4.17)
Cannulation time, min	18.98 ± 4.10
Cannulation success	46 (95.83)
Atherosclerosis	1 (2.08)
Dissection	1 (2.08)
Lower-limb ischemia	2 (4.17)
Hematoma	2 (4.17)
Incision infection	0 (0.00)
Survival cases	35 (72.92)

Values are expressed as n (%) or mean±standard deviation. *PTA*, Posterior tibial artery.

#### Comparison of retrograde and antegrade distal perfusion

Retrograde distal perfusion was compared with antegrade distal perfusion (including percutaneous and surgical cannulation). For age, which conformed to a normal distribution, one-way analysis of variance was used. For body weight, which did not conform to a normal distribution, the Kruskal-Wallis test was applied. No statistically significant differences were observed in gender, age, body weight, or disease type among the three groups (all *P* > .05). The baseline data were generally balanced, indicating that the groups were comparable (see Table 2).

**Table 2:** Comparison of baseline data

Variable	Group				
	Retrograde	Antegrade percutaneous	Antegrade surgery	Statistical values	*P* value
Cases, n	48	93	23	164	
Gender, n					
Male	34	61	16	χ^2^ = 0.441	.802
Female	14	32	7		
Age, y, mean ± SD	49.48 ± 15.47	53.63 ± 10.28	48.21 ± 14.91	F = 2.688 (ANOVA)	.071
Body weight, kg, mean ± SD	68.99 ± 10.92	70.61 ± 13.31	69.13 ± 10.70	H = 0.757 (Kruskal-Wallis)	.685
Diagnosis, n					
Cardiomyopathy	15	20	5	χ2 = 2.586	.629
AMI	22	54	14		
Others	11	19	4		

*SD*, Standard deviation; *ANOVA*, analysis of variance; *AMI*, acute myocardial infarction.

Statistically significant differences were observed among the 3 groups in terms of primary cannulation success and infection rates. Further pairwise comparisons revealed that the retrograde DPC group achieved a significantly greater cannulation success rate compared with the antegrade percutaneous group (95.83% [46/48] vs 53.76% [50/93], *P* < .05), while demonstrating a lower infection rate than the antegrade surgical group (0.00% [0/48] vs 34.78% [8/23], *P* < .05). No statistically significant differences were found in the incidence of limb ischemia, hematoma formation, or survival rates among the groups (see Table 3).

**Table 3:** Comparison of the 3 distal perfusion approaches

Variable	Group				
	Retrograde	Antegrade percutaneous	Antegrade surgery	Statistical values	*P* value
Cases, n	48	93	23	164	
Primary cannulation success, % (n)	95.83% (46)	53.76% (50)	100.00% (23)	χ^2^ = 38.260	<.001
Lower-limb ischemia, % (n)	4.17% (2)	8.60% (8)	0.00% (0)	Fisher exact test	.348
Hematoma, % (n)	4.17% (2)	13.98% (13)	21.74% (5)	χ^2^ = 5.122	.77
Infection, % (n)	0.00% (0)	3.23% (3)	34.78% (8)	Fisher exact test	<.001
Survival, % (n)	72.92% (35)	69.89% (65)	52.17% (12)	χ^2^ = 3.344	.188

## Discussion

Femoral artery cannulation represents the most common approach in VA-ECMO. However, the incidence of ALI can reach 10% to 70%.^3^^,^^5^ The occurrence of ALI constitutes a “second hit”^6^ in severe cases, which significantly increases mortality and impairs the quality of life in survivors. Indwelling a DPC in the ipsilateral limb is an effective strategy to address this complication,^7^ as it can compensate for the insufficient distal tissue perfusion caused by femoral artery cannulation. Prophylactic insertion within 6 hours after ECMO initiation is recommended.^8^^,^^9^ Although the DPC cannot completely prevent the occurrence of ALI, it can reduce its incidence to 6.5% to 8.6%.^10^ The most used approach is antegrade DPC placement in the ipsilateral SFA. This can be achieved percutaneously; however, due to distal vascular collapse, this procedure is prone to failure and often requires multiple repeated punctures. Alternatively, surgical cannulation can be employed, but this approach involves complex procedures and carries risks such as major bleeding.

In 2012, Spurlock and colleagues^4^ first proposed the method of retrograde distal perfusion via a DPC placed in the PTA. However, this approach has not been widely adopted, and relevant reports remain scarce. This may be attributed to the fact that most clinicians are either unfamiliar with this technique or skeptical about its perfusion efficacy. Through a study of 48 cases, the present research further confirms the feasibility of this perfusion method.

Cannulation via the PTA can be performed at the bedside. Given that the PTA is superficially located and accompanied by 2 concomitant veins, it is easy to locate and access. Nevertheless, the procedure requires a certain level of surgical expertise. Spurlock and colleagues^4^ recommended ligating the distal end of the PTA during cannulation and the proximal end during decannulation. However, in all 48 cases at our center, no ligation was performed at either site. Hemostasis was achieved solely by compression upon catheter removal. No bleeding, hematoma, or other vascular complications were observed, suggesting that vessel ligation may be unnecessary. Due to reduced or absent distal blood flow, backflow of blood may not be observed during cannulation. Therefore, it is highly necessary to routinely perform ultrasound examinations postoperatively to confirm that the catheter is located within the true vessel lumen. It has been reported that the retrograde perfusion flow rate needs to be maintained between 100 and 300 mL/min.^4^ In the present study, the average perfusion flow rate was approximately 100 mL/min, which fully met the perfusion requirements in all cases. In addition, both Doppler ultrasonography and angiographic examinations confirmed that blood flow could retrogradely reach the SFA, DFA, and other major vessels, adequately demonstrating the feasibility of retrograde distal perfusion. Among the 48 patients, 2 still developed lower extremity ischemia. The team promptly removed the ECMO arterial cannula, repositioned it to the contralateral side, and repaired the femoral artery to restore normal blood flow, resulting in improvement of the ischemic condition. Whether retrograde or antegrade distal perfusion is used, timely vascular repair to restore native blood flow is crucial when recurrent limb ischemia occurs.^11^

Numerous studies have confirmed that the non-use of a DPC is an independent risk factor for lower limb ischemia.^12^, ^13^, ^14^ Although antegrade distal perfusion remains the mainstream approach, comparative data indicate that retrograde distal perfusion is equally effective in reducing the incidence of limb ischemia and offers distinct advantages, including a greater cannulation success rate and a lower incidence of infection. Retrograde DPC cannulation is not affected by factors such as nonpulsatile blood flow or obesity, is relatively easy to perform, and provides effective distal perfusion. It may serve as a valuable supplementary approach for limb perfusion.

## Conclusions

In VA-ECMO, retrograde distal perfusion via the PTA is feasible and effective. The perfused blood can retrogradely reach vessels such as the SFA and DFA, thereby alleviating lower-limb ischemia. However, as a retrospective single-center study, the sample size remains limited, which constitutes a certain limitation. Further studies with an expanded sample size are still required to explore this approach in greater depth.

## Conflict of Interest Statement

The authors reported no conflicts of interest.

The *Journal* policy requires editors and reviewers to disclose conflicts of interest and to decline handling or reviewing manuscripts for which they may have a conflict of interest. The editors and reviewers of this article have no conflicts of interest.
